# Starting to Gel: How Arabidopsis Seed Coat Epidermal Cells Produce Specialized Secondary Cell Walls

**DOI:** 10.3390/ijms16023452

**Published:** 2015-02-04

**Authors:** Cătălin Voiniciuc, Bo Yang, Maximilian Heinrich-Wilhelm Schmidt, Markus Günl, Björn Usadel

**Affiliations:** 1 Institute for Bio- and Geosciences (IBG-2: Plant Sciences), Forschungszentrum Jülich, 52425 Jülich, Germany; E-Mails: catalin.voiniciuc@gmail.com (C.V.); m.schmidt@fz-juelich.de (M.H.-W.S.); m.guenl@fz-juelich.de (M.G.); 2 Institute for Botany and Molecular Genetics (IBMG), RWTH Aachen University, 52056 Aachen, Germany; E-Mail: boyang8635@gmail.com

**Keywords:** arabidopsis, seed coat epidermis, cell wall biosynthesis, mucilage, pectin, hemicellulose, cellulose, glycosyltransferase, glycosyl hydrolase, transcription factor

## Abstract

For more than a decade, the Arabidopsis seed coat epidermis (SCE) has been used as a model system to study the synthesis, secretion and modification of cell wall polysaccharides, particularly pectin. Our detailed re-evaluation of available biochemical data highlights that Arabidopsis seed mucilage is more than just pectin. Typical secondary wall polymers such as xylans and heteromannans are also present in mucilage. Despite their low abundance, these components appear to play essential roles in controlling mucilage properties, and should be further investigated. We also provide a comprehensive community resource by re-assessing the mucilage phenotypes of almost 20 mutants using the same conditions. We conduct an in-depth functional evaluation of all the SCE genes described in the literature and propose a revised model for mucilage production. Further investigation of SCE cells will improve our understanding of plant cell walls.

## 1. Introduction

All growing plant cells are surrounded by a flexible extracellular matrix, called the primary cell wall, which is composed mainly of complex polysaccharides and proteins [1,2]. The primary wall is essential for plant growth and is continuously remodeled during cell expansion. After fully expanding, some specialized cell types are reinforced with very thick secondary walls [1,2]. The fibrous polymers found in plant secondary walls are used for the production of a variety of commodities including lumber, textiles, thickeners and biofuels [2,3]. The availability of the *Arabidopsis thaliana* genome sequence and the isolation of mutants with cell wall defects has provided an excellent platform to explore polysaccharide biosynthesis and modification [4]. For example, forward and reverse genetic studies of xylem formation led to the discovery of many important secondary wall genes [5,6]. Despite this progress, the majority of genes predicted to encode cell wall-related proteins remain to be functionally characterized. The seed coat epidermis (SCE) provides an alternative system to investigate the production of cell wall polysaccharides, particularly the synthesis and modification of pectin [7,8,9,10,11].

## 2. The Development of Seed Coat Epidermal Cells

### 2.1. Specialized Wall Production

**Figure 1:**
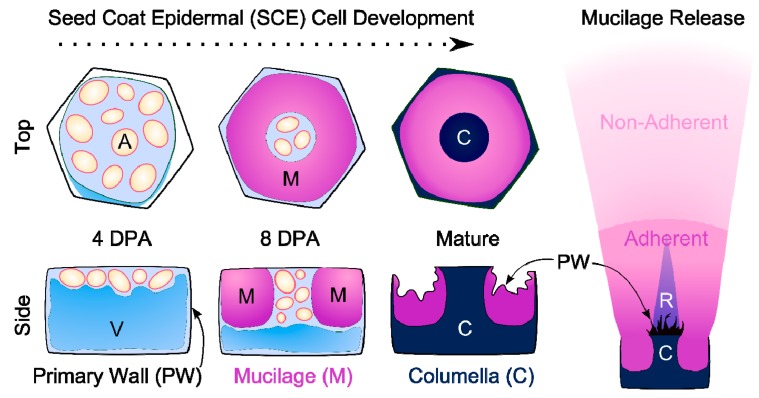
Models of SCE cell wall deposition and extruded mucilage structure. Young cells contain amyloplasts (A) and a large vacuole (V). Each SCE cell produces a mucilage ring, followed by a columella (C). Hydration of dry seed makes the mucilage burst out, rupturing the primary wall and forming a two-layered capsule and a ray-like structure (R).

Histological studies of seed coat development revealed that SCE cells synthesize a primary wall and two distinct secondary walls [12,13,14]. Figure 1 illustrates the sequential deposition of these walls. By four days post-anthesis (DPA), SCE cells have fully expanded and have accumulated amyloplasts, which partially displace the central vacuole (V). From five to eight DPA, the cells synthesize mucilaginous polysaccharides and secrete them in a polar manner [13,15]. The deposition of a thick ring of mucilage results in the formation of a volcano-shaped cytoplasmic column. From nine to 13 DPA, the cytoplasmic column is gradually displaced by the deposition of a secondary wall known as the columella [13,15]. By 18 DPA, the SCE cells reach maturity and are fully desiccated [13].

Mucilage is stored in the SCE until the mature seed is hydrated. Upon contact with water, mature Arabidopsis seeds quickly release copious amounts of mucilage from SCE cells. This was first exploited in a forward genetic screen for *mucilage-modified* (*mum*) mutants [16]. Although mucilage is dispensable in the laboratory [11], its adhesive properties and its ability to retain water may play important roles in facilitating seed dispersal and germination; see recent reviews [11,17].

### 2.2. Covered by a Cuticle?

Transmission electron microscopy (TEM) images of developing SCE cells indicate that a densely-stained cuticle-like structure covers the outer primary cell wall [18,19]. This may explain why the developing SCE cells are hydrophobic and could not be treated with some commonly used stains and drugs [20]. However, two other studies provide histological, chemical and genetic evidence of a cuticle in the inner seed coat [12,21]. Therefore, the number and location of cuticles in the Arabidopsis seed coat remains unclear.

## 3. Mucilage Composition: More than Just Pectin

Extruded mucilage forms a gelatinous capsule around the seed, which can be directly stained or extracted for chemical analysis [10]. The extruded mucilage is not homogenous but can be separated into a non-adherent capsule and an adherent layer. The non-adherent capsule represents around 65% of total mucilage and is easily detached from the seed [13,22,23,24]. The remaining 35% of mucilage forms an adherent layer that is difficult to remove from the seed, even with harsh chemical treatments [25]. This inner layer is associated with ray-like structures that radiate outwards from the cell wall fragments atop each columella (Figure 1) [22,23,24]. Several reviews have already addressed the structure, properties and function of rhamnogalacturonan I (RG I), the main component of both mucilage layers [7,8,9,10,11]. This review aims to highlight that Arabidopsis seed mucilage is more than just pectin, and that the structures of its minor components play equally important roles in controlling mucilage properties. Indeed this is not surprising, as other plant species have seed mucilage that is primarily composed of cellulose, heteromannans, or arabinoxylans; reviewed in [11]. Moreover, Arabidopsis mutants affecting cellulose and hemicellulose biosynthesis were shown to have defects in mucilage properties [22,23,24,26].

Whilst it is promising to investigate the precise role of these minor components, the analysis of mucilage composition can be hindered by a number of factors. The structure and properties of mucilage may be influenced by biotic and abiotic stress, as well as earlier defects in plant development. Even the manner in which seeds are harvested from a single plant might have an effect, as mucilage production may differ between primary and secondary inflorescences [27]. Since Arabidopsis mucilage has been extracted with a variety of solvents, shaking intensities and temperatures (summarized in Table S1), comparing the precise amounts of its components detected in different research articles is challenging.

**Figure 2:**
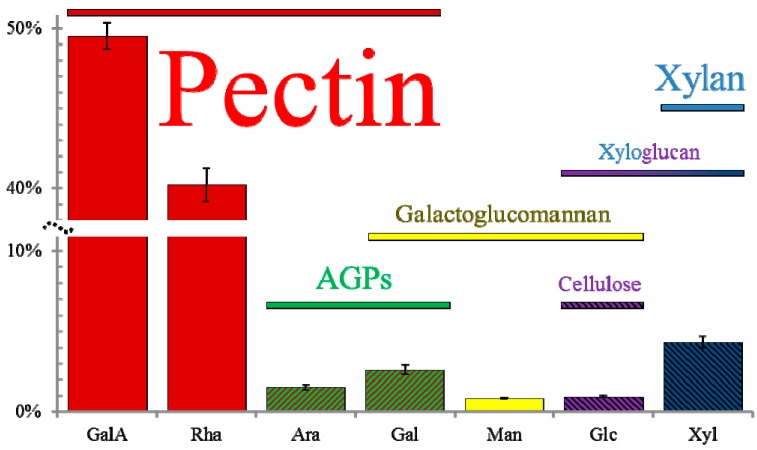
Molar percentage (mol %) of monosaccharides in mucilage and predicted polymers. Total mucilage was extracted by vigorously shaking Col-0 wild-type seeds for 30 min at 30 Hz. Values represent the mean ± SD of six biological replicates. The colored horizontal bars indicate the monosaccharides that are the building blocks of particular cell wall polymers. The font sizes reflect the estimated abundance of each mucilage polymer, except for “Pectin”, which has been scaled down by around 700% to fit in the figure.

To investigate the non-pectic components of mucilage, we carefully re-assessed the chemical composition of wild-type seed mucilage using data from many independent studies [20,24,25,28,29,30,31,32,33,34,35,36,37]. We compiled the mucilage monosaccharide composition and glycosyl linkage data from more than 20 publications in a single Excel file (Table S1), which provides a convenient platform to analyze the published datasets. The previously published results listed in Supplemental Table S1 are generally consistent with our own quantification of Columbia-0 (Col-0) wild-type mucilage composition (Figure 2, Table S3). The precise plant growth conditions, seed mucilage extraction, and analytical techniques we used are described in the Supplemental Methods file.

In addition to large amounts of rhamnose (Rha) and galacturonic acid (GalA), Arabidopsis seed mucilage extracts typically contain five additional monosaccharides (hereafter collectively referred to as the minor sugars): arabinose (Ara), galactose (Gal), glucose (Glc), xylose (Xyl) and mannose (Man); see Figure 2. Two other monosaccharides were also detected in some mucilage extractions, but both were even less abundant than the five minor sugars. Glucuronic acid (GlcA) was only reported in three studies, and was found to represent below 0.5 mol % of Col [30,33], and Wassilewskija (Ws) wild-type mucilage [38]. Fucose (Fuc) was more frequently detected but was always present only in trace amounts [28,30,32,35].

### 3.1. Pectins

#### 3.1.1. Rhamnogalacturonan I

Seed mucilage is readily stained by ruthenium red (RR), a dye that binds acidic biopolymers such as pectin [39]. Chemical analyses of Arabidopsis mucilage extracts revealed that Rha and GalA are the most abundant monosaccharides in both mucilage layers (Figure 2) [13,25]. Multiple studies found that around 90% of wild-type mucilage is composed of RG I, which has a backbone of alternating 4-GalA and 2-Rha linked residues [26,30,32,33,35]. While most of the RG I backbone is unbranched, some Rha residues can be substituted with Ara or Gal residues [30,31,38]. The abundance of RG I in mucilage is also supported by the observation that a mutant lacking a UDP-Rha synthase has a severe reduction in both GalA and Rha [29,40]. This observation is further corroborated by the fact that mucilage in developing SCE cross-sections was strongly labeled by CCRC-M36 [15], INRA-RU1 and INRA-RU2 [41] monoclonal antibodies that specifically bind to unbranched RG I epitopes.

#### 3.1.2. Homogalacturonan

Smaller quantities of another pectin polymer, homogalacturonan (HG), have also been detected in mucilage by immunolabeling of whole seeds with monoclonal antibodies [20,25,42]. HG is an unbranched polymer made of repeating 4-GalA residues, many of which are esterified with methyl or acetyl groups [20,36,42,43,44]. The degree of methylesterification (DM) and the degree of *O*-acetylation (DAc) control the ability of pectin polymers to form gels via calcium cross-links, and may play a role in preventing microbial degradation [45,46,47]. The DAc of Arabidopsis seed mucilage has not been widely examined, but is reported to be 5–14 mol % of uronic acids [44]. The DM of mucilage has been characterized in greater detail. Several research groups detected methanol release after alkaline de-esterification of mucilage extracts [16,20,26,36,43,48], and the DM of mucilage was estimated to be 30–34 mol % of uronic acids [20,26]. Complementary experiments showed that wild-type mucilage extruded in water can be labeled with JIM5 and JIM7 monoclonal antibodies directed against methylesterified HG [20,25,36,42,43], but not with 2F4 and PAM1 antibodies [20,42], which require long stretches of unesterified HG [42,49]. However, PAM1 can bind to wild-type mucilage de-esterified using sodium carbonate [42]. The *fly1* mutant has mucilage with a reduced DM, and displays delayed mucilage release and the detachment of many primary wall disks [20]. 2F4 labeled both the detached primary wall disks and the underlying mucilage columns in the *fly1* mutant [20]. Two other mucilage mutants with a low DM, *pmei6* and *sbt1.7*, display severely impaired mucilage extrusion and the detachment of outer primary walls as large sheets [36,43]. JIM5 and JIM7 only labeled the primary wall sheets in *pmei6* [36], but labeled both the sheets and the underlying mucilage in *sbt1.7* [43]. Therefore, wild-type mucilage must itself contain some methylesterified HG, although many epitopes detected by immunolabeling may be derived from primary wall fragments.

### 3.2. Hemicelluloses

#### 3.2.1. Galactoglucomannan

The building block of heteromannans, 4-linked Man, has been consistently reported in Arabidopsis seed mucilage [26,30,32,33,35,38], and represents 1.3–4.3 mol % of 2 M NaOH-extracted mucilage [26,32]. The 4,6-Man residues, characteristic of substituted mannans, are detected in most mucilage studies [26,32,35,38], and can be as high as 3.8 mol % of 2 M NaOH-extracted mucilage [32]. This indicates that mucilage may contain highly substituted heteromannans. LM21, a monoclonal antibody that binds equally to mannan, glucomannan and galactomannan [50], can strongly label wild-type extruded mucilage, including the ray-like structures depicted in Figure 1 [26]. The *csla2* mutant [26], which lacks a known glucomannan synthase [51], shows reduced LM21 mucilage labeling, reduced Man linkages, as well as fewer 4-linked Glc and terminal Gal residues [26]. This suggests that mucilage may contain galactoglucomannan [26], a polymer primarily made of Man, whose backbone also contains Glc, and can be frequently substituted by Gal [52]. The presence of this polymer in mucilage has not been confirmed yet and requires further investigation.

#### 3.2.2. Xylans

The xylan backbone consists of repeating 4-Xyl residues, which have been consistently detected in mucilage [26,30,32,33,35,38], and represent 3.4–5.3 mol % of 2 M NaOH-extracted mucilage [26,32,38]. The presence of xylans in mucilage has not been comprehensively tested by immunolabeling, and so far no direct evidence for xylans in mucilage has been found. Extruded mucilage could not be labeled with the LM10 monoclonal antibody [15], which is specific to unsubstituted or low-substituted xylans [53]. This however, could be due to masking effects of the LM10 epitope in mucilage, or to the fact that xylan in mucilage exhibits an unusual structure that might not be recognized by this antibody. Indeed, most publications report linkages suggestive of branched xylans (2,4-Xyl represents 0.4–1.9 mol % of 2 M NaOH-extracted mucilage [26,32,38]), however the nature of these side chains cannot be deduced from the available literature data. In Arabidopsis, xylans are typically substituted with single GlcA units [54]. However, GlcA cannot fully account for xylan branches in mucilage because it is either detected only in trace levels [30,33,38], or not at all [26,28,32,35].

#### 3.2.3. Xyloglucan

Another Xyl-containing hemicellulose could also be present in mucilage (Figure 2). Several articles state that in addition to pectin and cellulose, Arabidopsis mucilage contains xyloglucan [8,22,55]. Xyloglucan has a backbone made of 4-Glc residues, which are frequently branched at the O-6 position with terminal Xyl or 2-Xyl that is further substituted [52,56]. Such linkages are indeed present in mucilage [26,28,30,32,33,35,38], but most of them are not unique to only one polymer. In 2 M NaOH-extracted mucilage, 4,6-Glc represents 1.3–2.9 mol % [26,32,38], while 2-Xyl equals 0.6 mol %, but was only detected in two studies [32,38]. Epitopes recognized by an anti-xyloglucan (α-XG) polyclonal antibody were found in the primary walls, mucilage, and columella of SCE cells [15]. The labeling of the SCE secondary walls with α-XG is intriguing [15], since xyloglucan is typically the most abundant hemicellulose in primary walls and is replaced by xylans and mannans in secondary walls [52]. While α-XG is specific for xyloglucan when tested against pectin and extensin [57], its cross-reactivity with other hemicelluloses has not been examined. Considering that 50% of Glc in mucilage extracts may be derived from cellulose polymers (see following section) [55], xyloglucan is likely to be less abundant than mannans and xylans in mucilage.

### 3.3. Cellulose

Cellulose is composed exclusively of 4-Glc linkages and appears to be a critical component of mucilage. SCE cells lacking the CESA5 cellulose synthase subunit produce mucilage that is less adherent to the seed than in the wild type [22,23,24]. A detailed chemical analysis of *cesa5* seeds found changes consistent with 50% lower cellulose levels in three different experiments [55]: monosaccharide composition of water-extracted mucilage, seed crystalline cellulose quantification using the Updegraff method [58], and whole seed monosaccharide composition. Crystalline cellulose microfibrils in extruded mucilage are observed to cause birefringence of polarized light [23,26,59]. Extruded mucilage labeled with glucan-binding antibodies or dyes displays ray-like structures that extend from the top of columellae to the edge of the adherent mucilage capsule (Figure 1) [20,22,23,24,25,55,60]. One caveat of these studies is that the probes used may also recognize non-cellulosic polysaccharides. For example, Pontamine Scarlet 4B mainly labels cellulose, but can also bind to xyloglucan [61]. CBM3a, a carbohydrate-binding module directed against crystalline cellulose, exhibits a broad specificity for cell wall binding [60]. Paradoxically, CBM3a binds more to *cesa5*, which should have decreased crystalline cellulose levels, than wild-type mucilage [23,55]. Potentially, CBM3a cellulose epitopes in the wild type could be masked by pectin polymers [23], or CBM3a might also bind other wall polymers that are more abundant in *cesa5*.

### 3.4. Proteins

Proteins typically only represent a small fraction of the plant cell wall composition but play essential roles in its function [2]. Indeed, small amounts of proteins have been detected in mucilage [25]. Mucilage likely contains arabinogalactan proteins (AGPs), which are heavily glycosylated with Gal and Ara residues [62]. All the linkages representative of type II AGPs are typically found in mucilage extracts: 3-Gal, 6-Gal, 3,6-Gal, 3-Ara and 5-Ara [26,28,30,32,33,35]. The AGP known as SALT-OVERLY SENSITIVE5 (SOS5) may be a mucilage component since it promotes mucilage adherence to the seed [24,55].

In addition to structural proteins, mucilage likely contains several extracellular enzymes that modify the structure of its components [30,31,36,37,38,43]. The exact location of most of these enzymes in SCE cells is unknown. The roles of these proteins in mucilage maturation are described in Section 6.

## 4. Regulation of SCE Development

The analysis of transcript levels during seed coat development indicates that distinct genes are likely required for the production of the two specialized secondary walls in SCE cells [63]. We surveyed the expression profiles of more than 35 genes (see Table S2), which are reported to affect mucilage properties in the literature, and found that only a few of them are specifically associated with mucilage production in two independent seed coat microarrays [63,64,65]. In the following subsections we discuss the upstream regulators of SCE differentiation, while the enzymes and proteins that are more directly involved in cell wall production and modification are described in Section 5 and Section 6.

### 4.1. Outer Integument Establishment

The correct establishment of the inner and outer ovule integuments is essential for normal seed coat development to proceed. Three transcription factors (TFs): APETELA2 (AP2) [16,63,66], and two functionally redundant NAC family proteins (NARS1 and NARS2) [67] were shown to be positive regulators of outer seed coat differentiation. Mutants affecting these genes lack the early developmental cues required for SCE differentiation, and do not produce secondary walls.

Despite not being mentioned in previous mucilage studies, several other TFs are also known to maintain the identity of the outer integument and to promote its growth [68]. Two functionally redundant KANADI family members (KAN1 and KAN2), a YABBY protein (INNER NO OUTER, INO), and a class III homeodomain leucine zipper (HD-ZIP III) protein (REVOLUTA, REV) control outer integument formation, and their absence causes amorphous or arrested growth [68,69]. Another KANADI protein, KAN4, maintains the identity of the inner ovule integument [68]. The role of *KAN4* in ovule development was identified by map-based cloning of the *aberrant testa shape* (*ats*) mutation [69], which causes abnormal seed shape and was reported to “produce very little mucilage” [70]. Despite their altered shape, *ats* seeds display normal SCE cell wall surface morphology in scanning electron micrographs of two independent studies [69,70], unlike mutants such as *ap2*, *ttg1*, and *gl2* mutants that have reduced mucilage and columella production [16,66,70,71]. It is therefore likely that the *ats* mutant exhibited pleiotropic extrusion defects, despite producing sufficiently large amounts of mucilage to generate normal columella shape. Contrary to current genetic models for SCE development [9,11], ATS is likely not a key regulator of the outer seed coat layers.

### 4.2. Control of SCE Differentiation

NARS1 and NARS2 may have partial functional overlap with TTG2 in SCE development [67], and were recently found to be positive regulators of PER36, an important regulator of mucilage release [37]. However, the *per36* mutant does not explain the loss of secondary walls in the *nars1 nars2* double mutant, so additional genes involved in mucilage production must be regulated by these TFs.

**Figure 3:**
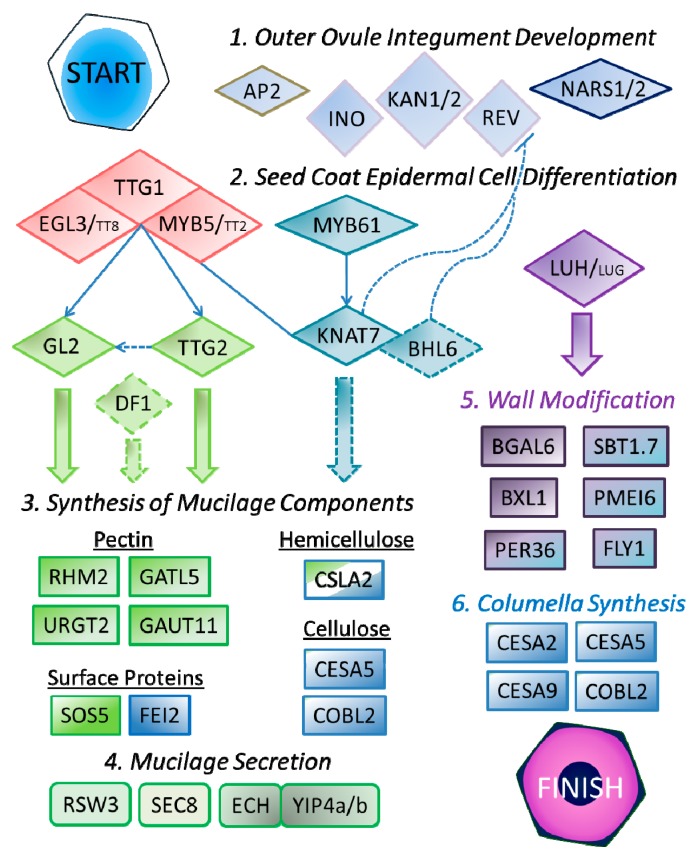
A simplified model of SCE development showing transcription factors (diamonds) and enzymes or other proteins (rectangles) involved in cell wall production. Slashes denote functionally redundant homologs, and colors highlight proteins with similar functions. Overlapping shapes form a protein complex. In addition, KNAT7 can directly interact with MYB5. Thin arrows indicate direct transcriptional activation, while dashes show putative interactions that have not been validated in SCE cells.

TTG1 is a WD40 repeat protein that regulates several biochemical pathways in Arabidopsis [72]. In addition to controlling anthocyanin production, the development of trichomes and root hairs, TTG1 is an essential positive regulator of seed mucilage synthesis [72]. Throughout the plant, TTG1 functions as part of a complex with MYB and bHLH TFs [73,74]. For the regulation of mucilage production, TTG1 interacts primarily with MYB5 and the bHLH protein EGL3 [71,74]. MYB5 and EGL3 show partial functional redundancy with TT2 and TT8, respectively, which are important TFs for anthocyanin synthesis [71,73,74,75]. While *tt2* and *tt8* single mutants have normal mucilage phenotypes, *tt2 myb5* and *egl3 tt8* double mutants show more severe mucilage defects than the *myb5* and *egl3* single mutants [71,74]. As shown in Figure 3, the TTG1-bHLH-MYB complex directly regulates the expression of GL2 and TTG2 [74,76,77]. *RHM2* expression, which is essential for RG I synthesis, is regulated by GL2, but not TTG2 [40]. TTG2 may positively regulate *GL2* levels in epidermal cells [73], and both are equally important for expression of *GATL5*, another gene involved in pectin synthesis [35].

Mucilage production is also controlled by DF1, a trihelix protein whose downstream targets and relation to other seed coat TFs are currently unknown [34]. Based on based on our re-assessment of a *df1* mutant alongside other mucilage TF mutants (see Section 7), we suggest that DF1 may function downstream of TTG2 in the mucilage production pathway (Figure 3). This hypothesis should be tested by analysis of *DF1* transcript levels in mutants affecting TTG2, GL2 and upstream TFs.

**Figure 4:**
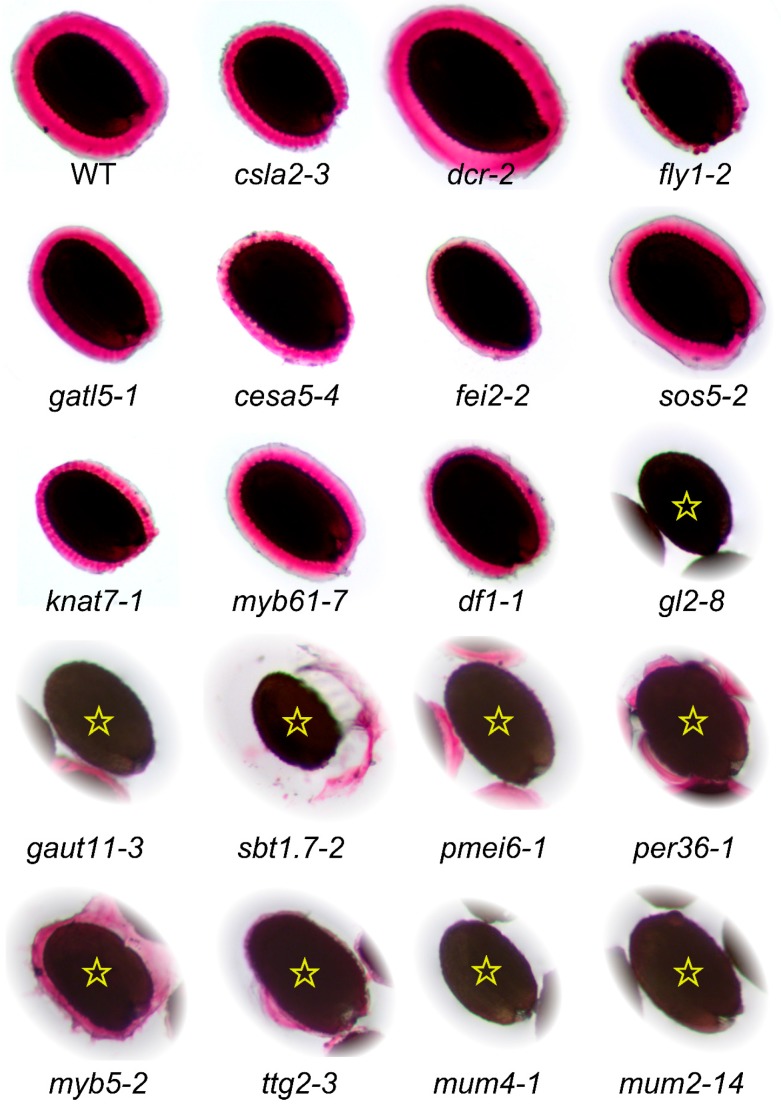
Analysis of RR staining phenotypes of WT and 19 known mucilage mutants. Seeds, grown under continuous light, were gently mixed with water for 5 min in a 24-well plate. The adherent mucilage was stained with 0.01% RR for 5 min. For the last nine mutants (marked with a star), most seeds float in the center of each well.

MYB61 appears to regulate cell wall synthesis in SCE cells via a distinct pathway, and is not as critical for mucilage production as TTG1 [28]. MYB61 was shown to bind the transcriptional regulatory region of *KNAT7* [78], a negative regulator of secondary wall formation [79]. KNAT7 can physically interact with MYB5 and MYB75/PAP1, which is involved in anthocyanin production [80]. While *myb75* does not have clear mucilage defects, *myb61* and *knat7* both have smaller adherent mucilage capsules compared to WT (see Figure 4) [28,78,80]. Our re-analysis of *myb61* and *knat7* indicates that they produce total mucilage levels similar to wild-type, but have reduced amounts of minor components that could be part of hemicellulose (discussed in Section 7). Recently, KNAT7 and BEL1-LIKE HOMEODOMAIN6 (BLH6) were shown to form heterodimers that regulate secondary cell wall synthesis in the Arabidopsis stem via repression of *REV* [81]. KNAT7 and BLH6 may also be involved in repressing *REV* in the seed coat (Figure 3). Consistent with a role in mucilage production, *BLH6* expression peaks at 7 DPA during seed coat development in two microarrays [63,64,65], but the *blh6* mucilage phenotype has not yet been described.

**Figure 5:**
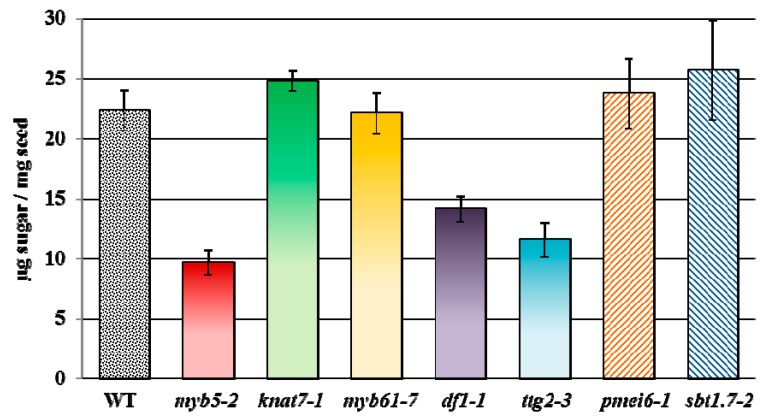
Total mucilage monosaccharides extracted from WT and several mutants. Values represent the mean ± SD of three biological replicates for each mutant and six for the WT. Total mucilage was extracted by vigorously shaking 5 mg seeds in 1 mL water using a Retsch Mill (30 Hz for 30 min). Monosaccharides obtained by trifluoroacetic acid hydrolysis were quantified using ion chromatography (see Supplemental Methods for a detailed protocol)

The modification of mucilage is promoted by MUM1*/*LUH, and is not controlled by TTG1-related TFs [32]. In developing leaves, LEUNIG (LUG) and LEUNIG_HOMOLOG (LUH) form a transcriptional repressor complex through physical interactions with SEUSS (SEU), SEUSS-LIKE (SLK), and YABBY proteins [82]. An early stop codon in the *LUH* coding sequence was shown to be responsible for the *mum1* mucilage defects [32,33]. Mutations in *SEU* do not affect mucilage extrusion, however this could be due to functional redundancy with three *SLK* genes that are expressed in the seed coat [33,83]. While *LUG* expressed under the control of the *LUH* promoter rescued the *luh* mucilage defects [33], the normal mucilage extrusion of *lug* mutants and available transcript data indicate that the *LUG* does not play a major role in mucilage synthesis and modification [33,83]. *MUM1/LUH* promotes *BGAL6*, *BXL1*, *SBT1.7* and* PMEI6* expression [32,36], although it remains unclear if this occurs via direct activation of its targets [32], or via repression of an intermediary inhibitor [33].

## 5. Genes Involved in Mucilage Production

### 5.1. Mucilage Synthesis

Since mucilage contains a mixture of polysaccharides, a complex set of genes encoding nucleotide sugars interconversion enzymes, nucleotide sugar transporters (NSTs) and glycosyltransferases (GTs) must be active in SCE cells. The UDP-l-Rha synthase RHM2/MUM4 catalyzes the conversion of UDP-Glc to UDP-Rha, a key substrate for RG I synthesis [29,40,84]. The *rhm2* seeds produce very little mucilage and have flattened columella. *RHM2* expression in the seed coat peaks at 7 DPA, consistent with its role in mucilage production [40,63].

Substrate availability for mucilage synthesis also depends on the function of NSTs. A recent study found that URGT2, a bifunctional UDP-l-Rha/UDP-d-Gal transporter, is responsible for transporting UDP-Rha from the cytosol into the Golgi lumen [85]. *URGT2* shows a seed coat expression pattern consistent with a role in mucilage synthesis, and its absence leads to reduced RG I production as determined by immunolabeling with CCRC-M36 and monosaccharide composition [85]. One *urgt2* allele also showed a significant reduction of Gal in mucilage, suggesting that URGT2 may also be responsible for UDP-Gal transport in SCE cells [85].

The analysis of galacturonosyltransferase (GAUT) and GAUT-LIKE (GATL) protein families has led to the identification of two GTs, GAUT11 and GATL5, which affect mucilage properties [35,86]. GAUT11 has not been characterized in detail but one *gaut11* allele previously showed impaired mucilage extrusion and 30% lower Rha and GalA levels compared to the wild type [86]. The loss of GATL5 causes a 40% decrease in mucilage Rha and GalA content, but surprisingly increases the molecular weight of mucilage by 60% [35]. The authors suggest that the role of GATL5 in mucilage production is more intricate than simple RG I backbone elongation [35]. The precise roles of GAUT11 and GATL5 in pectin biosynthesis require further investigation.

CSLA2, a member of the CELLULOSE SYNTHASE-LIKE A protein family, is involved in glucomannan synthesis in mucilage [26]. Mutations in *CSLA2* result in a more compact mucilage capsule, despite normal adherence. Interestingly, the loss of heteromannan in mucilage alters the spatial organization of mucilage cellulose and appears to cause a significant reduction of crystalline cellulose. This implies that heteromannan and cellulose cross-links modulate the ultrastructure of adherent mucilage [26].

CESA5 is important for cellulose production in the SCE, and mediates the adherence of the inner mucilage layer to the seed [22,23,24,55]. Since cellulose is synthesized by a rosette complex composed of different CESA subunits, other CESA proteins may be required for cellulose deposition in mucilage [22,23]. Although CESA2 and CESA9 do not affect mucilage properties, they are involved in columella formation together with CESA5 [22,87].

The loss of either the FEI2 receptor-like kinase or the SOS5 AGP also results in increased mucilage detachment [24,55,88]. FEI2 and SOS5 are more important for the formation of ray structures in extruded mucilage than CESA5 [24,55,88]. A recent study demonstrated that SOS5 and CESA5 facilitate mucilage adherence through distinct mechanisms [55], contrary to initial reports that both function in the same pathway [24,88]. SOS5 does not affect cellulose biosynthesis, but instead establishes pectin-based connections in seed mucilage.

A new study revealed that *COBRA-LIKE2* (*COBL2*) is co-expressed with *FEI2* during seed coat development and is essential for mucilage adherence and crystalline cellulose synthesis in the seed [59]. Two related proteins, COBRA and COBL6 do not affect cellulose deposition in the mucilage pockets [59]. Although the precise biochemical function of these proteins is unknown, they are proposed to facilitate the assembly of multiple 4-linked Glc chains into crystalline cellulose [59].

### 5.2. Polar Secretion of Mucilage

Non-cellulosic mucilage components are secreted to the apoplast in a polar manner, a process that typically occurs in cells undergoing tip growth [13]. Several players involved in the secretion of mucilage polysaccharides have been identified. RSW3 is the catalytic subunit of glucosidase II, which processes *N*-glycans that are essential for correct protein folding, and is required for normal cell wall deposition in many tissues [89]. In SCE cells, RSW3 may be required for the secretion of mucilage, or to process the Golgi enzymes that synthesize mucilage polysaccharides [89].

*SEC8* encodes a subunit of the exocyst, an important regulator of exocytosis, and is essential for normal mucilage production [27]. EXO70A1, another exocyst subunit, and an interacting protein, ROH1, might also play a role in the transport of cargo to the cell wall [27], but mutant analyses indicate that they are not as important for proper mucilage deposition as SEC8.

Recently, YPT/RAB GTPase Interacting Protein 4a (YIP4a) and YIP4b were shown to form a complex with ECHIDNA (ECH), a protein localized in the *trans*-Golgi network [90,91]. The *yip4a* *yip4b* double mutant and *ech* mutant have aberrant mucilage release because they accumulate mucilage in the vacuole and the ER rather than in the apoplast [90,91]. Transmission electron micrographs show that most secretory vesicles in *ech* SCE cells cannot reach or fuse with the plasma membrane [91].

## 6. Genes Involved in Mucilage Modification

After mucilage is synthesized, certain components are modified to ultimately facilitate extrusion. BGAL6 and BXL1 were shown to function as glycosyl hydrolases involved in trimming RG I galactan and arabinan side chains, respectively [30,31,38]. Despite normal mucilage deposition in developing seeds, *bxl1* mutants show patchy mucilage release [38], while *mum2* mutants do not extrude any mucilage in water [30,31]. The drastic impairment of mucilage extrusion due to increased RG I branching highlights the importance of this pectin modification for mucilage gelling properties [30].

The DM is another important property for gelling properties and is directly modified in the apoplast by pectin methylesterases (PMEs) [92]. PMEs de-esterify HG to produce negatively charged pectin regions that can form cross-links via Ca^2+^ ions. The spatio-temporal modification of pectin DM is controlled primarily by PMEs and their inhibitors (PMEIs), and is essential for promoting *Brassicaceae* testa rupture and seed germination [93,94]. Although at least seven PME genes are expressed in the seed coat at 7 DPA [48], it is still unclear which PME enzymes modify mucilage. Despite this, several regulators of mucilage DM have been identified: a subtilisin-like serine protease (SBT1.7), a PME inhibitor (PMEI6) and a transmembrane RING E3 ubiquitin ligase (FLY1) [20,36,43]. These three genes are highly expressed during mucilage deposition in developing seed coats, and their absence leads to a reduction of mucilage DM shown by immunolabeling or chemical analysis [20,36,43]. The *sbt1.7* and *pmei6* single mutants seeds typically float in water because they do not readily release mucilage [36,43]. After prolonged mechanical agitation, mucilage is released from the *sbt1.7* and *pmei6* mutants, along with a large sheet of outer primary walls [36,43]. *SBT1.7* and *PMEI6* are proposed to regulate different targets since the double mutant showed higher PME activity compared to the two single mutants [36]. Interestingly, only some *fly1* cells have delayed mucilage release and the mutant seeds are surrounded by many small discs, which are detached outer primary walls [20]. All of the *fly1* mucilage defects can be rescued by treatment with cation chelators [20]. While SBT proteins are known to be involved in PME processing and PMEIs can directly bind PMEs [92,95], FLY1 represents the first E3 ubiquitin ligase that specifically regulates the DM of pectin [20]. The exact proteins targeted by SBT1.7, PMEI6, and FLY1 are still unknown and require further investigation.

PER36, a member of the class III peroxidase family, is polarly deposited into the outer primary cell wall at the start of mucilage secretion and generates reactive oxygen species that may degrade RG I and/or HG [37]. The *per36* seeds show impaired mucilage defects that resemble the *sbt1.7* and *pmei6* mutants, and the detachment of sheets of primary walls after shaking [37].

## 7. Re-Assessment of Mucilage Defects

As discussed in the previous sections and summarized in Supplemental Table S2, more than 35 different loci are reported to affect seed mucilage properties. However, only small subsets of mucilage mutants have so far been described using the same growth conditions and staining procedures. To overcome this limitation, we grew homozygous mutants affecting 19 genes alongside Col-0 wild-type under continuous light (around 170 µE·m^−2^·s^−1^), constant temperature (20 °C) and relative humidity (60%); see Supplemental Methods for additional details. Insertional mutants were genotyped using the PCR “Touch-and-Go” method [96], with a reaction mix consisting of two gene-specific primers (Supplemental Table S4), and an appropriate T-DNA specific primer (see Supplemental Methods). The RR staining phenotype of these mucilage mutants is summarized in Table 1, and a representative seed for each genotype is shown in Figure 4. The vast majority of mutants analyzed, including the new alleles, show RR staining phenotypes consistent with previous publications. The *df1-1* seeds were originally described as not releasing any mucilage in water [34], but they can extrude a very small capsule of mucilage in our conditions (Figure 4). One notable exception is *dcr-2*, which causes cuticular defects throughout plant development and was reported to completely block mucilage release [19]. Under our growth conditions, we observed organ fusion defects in *dcr-2* leaves and inflorescences consistent with the previous publication [19], however the mutant seeds released mucilage upon contact with water and the stained capsule was not distinguishable from the wild type.

**Table 1:** A summary of mutants re-assessed for mucilage RR staining defects.

Mutant	Locus	Polymorphism	References	Mucilage RR Staining
*gatl5-1*	At1g02720	SALK_106615C	[35]	As previously described
*myb61-7*	At1g09540	SALK_106556C	[97]	Similar to [78], unlike [28]
*gaut11-3*	At1g18580	SAIL_567_H05	New allele	Similar to *gaut11-2* [86]
*mum4-1*	At1g53500	EMS mutation	[40]	As previously described
*knat7-1*	At1g62990	SALK_002098C	[80]	As previously described
*df1-1*	At1g76880	SALK_106258C	[34]	Very small capsule
*gl2-8*	At1g79840	SALK_130213C	[98]	As previously described
*fei2-2*	At2g35620	SALK_044226	[24,99]	As previously described
*ttg2-3*	At2g37260	SALK_148838	[77]	As previously described
*pmei6-1*	At2g47670	SM_3.19557	[36]	As previously described
*myb5-2*	At3g13540	SALK_105723C	[100]	Similar to *myb5-1* mucilage
*sos5-2*	At3g46550	SALK_125874	[24,55]	As previously described
*per36-1*	At3g50990	SAIL_194_G03	[37]	As previously described
*fly1-2*	At4g28370	SALK_067290	[20]	As previously described
*cesa5-4*	At5g09870	SALK_207154C	New allele	Similar defects to [22,23,24]
*csla2-3*	At5g22740	SALK_149092C	[26]	As previously described
*dcr-2*	At5g23940	SALK_128228C	[19]	Similar to wild-type
*mum2-14*	At5g63800	SALK_060221C	New allele	Similar defects to [30,31,97]
*sbt1.7-2*	At5g67360	GK-544E06.01	[43]	As previously described

In contrast to our results, the original study of *myb61* showed that mutant seeds did not release mucilage and ground seed extracts contained 50% fewer pectin monomers [28]. However, a recent study found that *myb61* mutants can release mucilage [78], consistent with our observations (Figure 4). While the first three *myb61* alleles are clearly described [28], we could not unambiguously determine the identity of the *myb61-4* and *myb61-5* alleles [78]. Since an EMS mutation in *MYB61* was called *myb61-6* [101], we describe the SALK_106556C insertion as *myb61-7*. The cause of the observed *myb61* phenotypic differences is unclear, although MYB61 is thought to be a pleiotropic regulator of resource allocation so it likely only indirectly affects seed mucilage production [78].

We also analyzed the mucilage monosaccharide composition of five known TF mutants (*myb5-2*, *knat7-1*, *myb61-7*, *df1-1* and *ttg2-3*), and two mutants that alter the DM of HG (*pmei6-1* and *sbt1.7-2*). Consistent with previous publications, *pmei6-1* and *sbt1.7-2* do not affect the total level of mucilage produced (Figure 5). Relative to the wild type, only three mutants have significant changes in the total amount of monosaccharides (*p*-value < 0.003, Student’s *t*-test). The *myb5-2*, *ttg2-3*, and *df1-1* mutants have mucilage reductions of 57%, 48% and 37%, respectively, compared to the wild type. Supplemental Table S3 shows the relative composition of monosaccharides in mucilage extracts from the TF mutants. While *myb61-7* and *knat7-1* release almost normal levels of mucilage, both mutants have significantly altered mol % of Xyl and Man (*p*-value < 0.006, Student’s *t*-test), which are reduced by 28%–43% and 15%–21%, respectively, compared to the wild type (Table S3). These observations suggest that *MYB61* and *KNAT7* may promote the synthesis of mucilage hemicellulosic components.

## 8. Conclusions

The hallmark of SCE cell differentiation is the sequential production of two specialized secondary walls: the mucilage ring and the columella. Our detailed re-analysis of mucilage biochemical data indicates that although Arabidopsis seed mucilage consists primarily of RG I, it likely also contains other polymers such as HG, substituted xylans, galactoglucomannan, xyloglucan, cellulose, and AGPs. Since the minor components of mucilage have been largely neglected, further studies must be conducted to elucidate the precise identity of hemicelluloses and apoplastic proteins in SCE cells.

Although genes affecting HG, cellulose, and glucomannan structure have gradually been identified, additional players must be involved in cell wall production and modification. Novel genes affecting cell wall structure will likely be discovered through genetic modifier screens [101], reverse genetic approaches [34,59], or by analyzing natural variation in mucilage properties [9,97]. The Arabidopsis SCE remains a rich resource for cell wall research that has not been fully exploited.

## Supplementary Materials

Supplementary materials can be found at http://www.mdpi.com/1422-0067/16/02/3452/s1.
